# Trends in Cervical Cancer Screening in Title X-Funded Health Centers — United States, 2005–2015

**DOI:** 10.15585/mmwr.mm6637a4

**Published:** 2017-09-22

**Authors:** Christina I. Fowler, Mona Saraiya, Susan B. Moskosky, Jacqueline W. Miller, Julia Gable, Nancy Mautone-Smith

**Affiliations:** ^1^RTI International, Research Triangle Park, North Carolina; ^2^Division of Cancer Prevention and Control, National Center for Chronic Disease Prevention and Health Promotion, CDC; ^3^Office of Population Affairs, U.S. Department of Health and Human Affairs; ^4^Office of Women’s Health, Health Resources & Services Administration.

Cervical cancer screening is critical to early detection and treatment of precancerous cells and cervical cancer. In 2015, 83% of U.S. women reported being screened per current recommendations, which is below the *Healthy People 2020* target of 93% (*1*,*2*). Disparities in screening persist for women who are younger (aged 21–30 years), have lower income, are less educated, are uninsured, lack a source of health care, or who self-identify as Asian or American Indian/Alaska Native (*2*). Women who are never screened or rarely screened are more likely to develop cancer and receive a cancer diagnosis at later stages than women who are screened regularly (*3*). In 2013, cervical cancer was diagnosed in 11,955 women in the United States, and 4,217 died from the disease (*4*). Aggregated administrative data from the Title X Family Planning Program were used to calculate the percentage of female clients served in Title X-funded health centers who received a Papanicolaou (Pap) test during 2005–2015. Trends in the percentage of Title X clients screened for cervical cancer were examined in relation to changes in cervical cancer screening guidelines, particularly the 2009 American College of Obstetricians and Gynecologists (ACOG) update that raised the age for starting cervical cancer screening to 21 years (*5*) and the 2012 alignment of screening guidelines from ACOG, the U.S. Preventive Services Task Force (USPSTF) and the American Cancer Society (ACS) on the starting age (21 years), screening interval (3 or 5 years), and type of screening test (*6*–*8*). During 2005–2015, the percentage of female clients screened for cervical cancer dropped continually, with the largest declines occurring in 2010 and 2013, notably a year after major updates to the recommendations. Although aggregated data contribute to understanding of cervical cancer screening trends in Title X centers, studies using client-level and encounter-level data are needed to assess the appropriateness of cervical cancer screening in individual cases.

The Title X Family Planning Program supports the delivery of contraceptive and related preventive care to a population that is predominantly female, low income, uninsured, young, and racially and ethnically diverse. For many clients, Title X centers are their only ongoing source of care. As a condition of their funding, Title X-funded health care providers are required to adhere to nationally recognized standards of care and adapt protocols as guidelines are updated. Among the 3.6 million female clients who received care in one of 3,900 Title X-funded health centers in 2015, more than 743,000 were screened for cervical cancer (*9*).

This analysis used data from the Family Planning Annual Report (FPAR), which is an annual reporting requirement for all Title X service grantees (*9*). The study examined FPAR data for 64 grantees in the 50 states and the District of Columbia that received continuous Title X funding during 2005–2015, a period during which the service networks for these grantees served 3.2 million to 4.3 million women annually (Table). For each grantee, an FPAR consists of aggregated data (e.g., client characteristics, services provided, and revenue) for all subrecipients and clinics that receive Title X funds.

**TABLE:** Characteristics of Title X grantees and demographic characteristics of female clients served — 2005–2015 Family Planning Annual Report,* 50 states and the District of Columbia

Characteristic	2005	2006	2007	2008	2009	2010	2011	2012	2013	2014	2015
**Network**											
Grantees (no.)	64	64	64	64	64	64	64	64	64	64	64
Subrecipients (no.)	1,045	1,062	1,054	1,046	1,036	1,012	1,036	1,040	1,067	1,030	1,098
Service sites (no.)	3,726	3,829	3,879	3,873	3,858	3,741	3,756	3,651	3,599	3,542	3,570
Females (millions)	4.14	4.16	4.15	4.18	4.27	4.25	4.08	3.93	3.76	3.43	3.22
**Age group (yrs)**											
≤19 (%)	26.5	26.2	25.5	25.0	23.9	22.4	21.1	19.8	18.5	18.4	18.1
20–24 (%)	32.5	32.3	31.8	31.3	31.1	31.3	30.6	29.9	29.4	28.8	27.8
25–29 (%)	18.3	18.8	19.4	19.7	20.1	20.6	21.1	21.6	22.0	22.1	22.2
≥30 (%)	22.7	22.7	23.3	23.9	25.0	25.7	27.2	28.7	30.1	30.6	31.9
**Race/Ethnicity; English proficiency**											
Black (%)	18.4	17.9	18.2	18.8	18.9	18.9	18.7	19.2	19.5	19.4	19.8
White (%)	64.9	66.1	63.4	59.7	59.4	58.1	57.1	56.4	55.9	55.2	54.4
Other (%)	6.5	6.5	6.8	7.3	8.0	9.4	9.9	10.4	9.0	8.6	8.3
Hispanic ethnicity (%)	21.2	22.3	24.0	25.2	25.5	26.3	26.7	27.3	28.4	29.3	31.0
LEP^†^ (%)	10.5	11.3	11.8	12.9	12.9	12.3	11.9	11.8	11.8	11.5	11.8
**Income (% PG)** ^†,§^											
≤100% (%)	65.7	66.6	68.9	69.7	69.3	68.4	68.1	70.9	70.2	68.6	67.3
101%–250% (%)	27.2	26.2	25.3	23.7	23.2	23.4	22.7	21.5	22.2	22.6	23.2
**Insurance** ^†^											
Uninsured (%)	61.1	62.6	66.0	65.8	65.6	66.2	63.9	65.0	63.1	54.2	47.7
Public (%)	20.8	20.9	21.2	21.6	19.9	23.0	25.2	23.3	24.7	29.4	35.5
Private (%)	7.7	8.5	9.0	9.4	8.4	8.7	8.7	9.4	10.0	13.7	15.2

**Abbreviations:** LEP = limited English proficiency; PG = poverty guideline.

* The Family Planning Annual Report (FPAR) is the annual reporting requirement of all Title X services grantees. FPAR data for Title X-funded centers are aggregated and reported at the grantee level. The study sample includes data for 64 grantees that received Title X funding during the entire study period; data for grantees in the U.S. Territories and Freely Associated States were excluded. https://www.hhs.gov/opa/title-x-family-planning/fp-annual-report/index.html.

^†^ Includes male clients.

^§^ Clients’ income is reported as a percentage of the U.S. Department of Health and Human Services poverty guideline for each year. https://aspe.hhs.gov/prior-hhs-poverty-guidelines-and-federal-register-references.

The outcome of interest was the percentage of female clients who received a Pap test. Because FPAR does not have Pap testing data by age or test type, age group–specific measures for receipt of other recommended preventive health services that are available in FPAR were included. These other preventive health service measures included the percentage of females aged ≤19 years and 20–24 years who were tested for chlamydia and the percentage of females aged ≤19, 20–29, and 30–44 years at risk for unintended pregnancy who adopted or continued using an effective contraceptive method. The inclusion of additional preventive care measures, particularly measures for females aged ≤19 years for whom cervical cancer screening was not recommended, permitted assessment of trends in other services that were expected to either increase or remain level. Females at risk for unintended pregnancy excluded those who were pregnant, seeking pregnancy, or not using a method for “other” reasons.* Effective contraceptive methods include female sterilization, vasectomy, intrauterine devices/systems; hormonal methods (implant, injectable, pill, ring, and patch); and diaphragm. Also included in the analysis was a measure for receipt of clinical breast exams; data on mammograms received were not available.

Trends in cervical cancer screening were compared with trends in the receipt of other recommended services to examine indirectly how changes in cervical cancer screening might reflect screening recommendations in effect during the analysis period. The expectations for the analysis were 1) a decline in cervical cancer screenings because of recommendations raising the starting age for screening and moving away from annual screenings; 2) no change or an increase in recommended chlamydia testing and contraceptive use; and 3) a gradual decline in clinical breast exams because of the differences in major recommendations about whether a clinical breast exam should be performed and the clarification in the *U.S. Selected Practice Recommendations for Contraceptive Use*^†^ that neither a Pap test nor a clinical breast exam contributes substantially to safe and effective contraceptive use.

During 2005–2015, the percentage of female clients screened for cervical cancer decreased gradually; the percentage of female Title X clients screened for cervical cancer declined from 51% in 2005 to 21% in 2015 (Figure). The largest 1-year decline (from 41% to 35%) occurred in 2010, after release of ACOG’s 2009 screening guideline that increased the recommended age for the first Pap test to 21 years. The second largest 1-year decline (from 27% to 23%) occurred in 2013, after the 2012 alignment of USPSTF, ACOG, and ACS recommendations on the age at first Pap test and age group–specific screening intervals.

**FIGURE:**
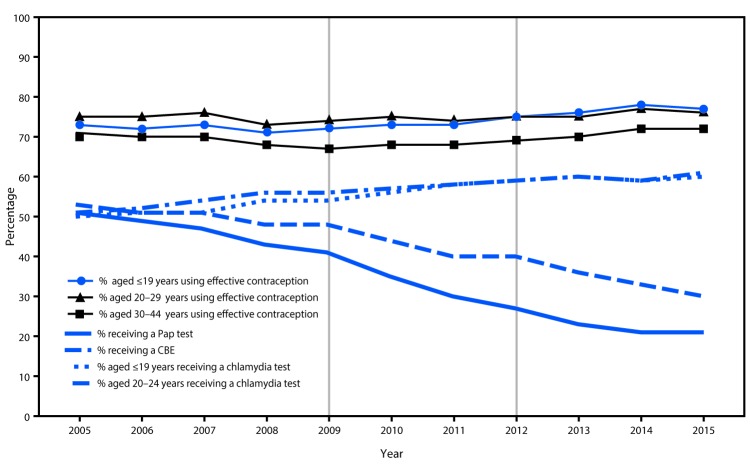
Cervical cancer screening recommendations in effect, including major changes in 2009 and 2012,* and percentages of female Title X clients in receipt of cervical cancer screening,^†^ chlamydia testing,^§^ and clinical breast exams,^¶^ and continued use or adoption of effective contraception** among, by year — Family Planning Annual Report,^††^ 50 states and the District of Columbia, 2005–2015 **Abbreviations:** ACS = American Cancer Society; ACOG = American College of Obstetricians and Gynecologists; CBE = clinical breast exam; USPSTF = U.S. Preventive Services Task Force. * During 2005–2012, cervical cancer screening recommendations from ACS, ACOG, and USPSTF for women at average risk with a cervix varied in terms of starting age (within 3 years of first sex or age 21 years), stopping age (65–70 years), and interval (annually, every 2 years, or every 3 years), based on age, prior negative test results, or type of screening test (conventional or liquid cytology or co-testing using a combination of cytology plus human papillomavirus DNA testing [HPV co-test]). During this period, there were two major changes in screening recommendations that are notable. In 2009, ACOG updated its cervical cancer screening recommendation by raising the starting age for screening to 21 years. In 2012, cervical cancer screening recommendations from ACS (March 2012), USPSTF (March 2012), and ACOG (November 2012) were congruent. The recommendations were that screening start at age 21 years, that it occur at the following intervals using specific methods; 21–29 years: every 3 years using cytology alone; 30–65 years: every 3 years (cytology) or every 5 years (HPV co-test); >65 years: stop screening if there is an adequate negative prior screening history, defined as two (co-test) or three (cytology) consecutive negative results within the past 10 years and the most recent test was performed within 5 years. https://www.cdc.gov/cancer/cervical/pdf/guidelines.pdf. ^†^ Percentage of females who received a Pap test in the calendar year. ^§^ Percentage of females aged ≤19 years or 20–24 years who received a chlamydia test in the calendar year. During 2005–2014, CDC recommended routine annual chlamydia screening for sexually active women aged ≤25 years and for sexually active older women at increased risk for infection (e.g., new or multiple partner[s]). In June 2015, CDC lowered the age range for routine annual screening to ≤24 years. During 2007–2015, the USPSTF recommended screening for sexually active women aged ≤24 years and for sexually active older women at increased risk for infection; evidence was insufficient to recommend an optimal screening interval. ^¶^ Percentage of females who received a CBE in the calendar year. During 2005–2015, ACOG recommended annual CBE for women aged ≥19 years and ACS recommended CBE with a periodic health exam every 3 years (aged 20–39 years) or annually (aged ≥40 years). In 2002, USPSTF concluded that evidence was insufficient to recommend for or against routine CBE alone to screen for breast cancer. In 2009, USPSTF concluded that current evidence was insufficient to assess the additional benefits and harms of CBE beyond screening mammography in women aged ≥40 years. ** Percentage of females aged ≤19, 20–29, and 30–44 years, at risk for unintended pregnancy (not pregnant or seeking pregnant, or not using method for “other” reason), who adopted or continued using effective contraception (female sterilization; vasectomy; intrauterine device; hormonal implant, injectable, pills, ring, or patch; and diaphragm) at their last encounter. ^††^ The Family Planning Annual Report is a reporting requirement of Title X service grantees. This study uses data for 64 grantees that received continuous funding during the study period. https://www.hhs.gov/opa/title-x-family-planning/fp-annual-report/index.html.

The percentage of clients receiving other recommended preventive health care, specifically chlamydia testing and contraception, increased or remained level, even in the 2 years (2010 and 2013) following major updates to cervical cancer screening recommendations (Figure). Among females aged ≤19 years for whom cervical cancer screening was not recommended by ACOG in 2009 or by USPSTF and ACS in 2012, the percentage tested for chlamydia increased from 54% (2009) to 60% (2015) and the percentage using an effective contraceptive method increased from 72% (2009) to 77% (2015). Among females aged 20–24 years, chlamydia testing rates increased from 56% in 2009 to 61% in 2015, and effective contraceptive use among females aged 20–29 years increased from 74% (2009) to 76% (2015). During 2005–2015, the percentage of females of all ages who received a clinical breast exam declined from 53% to 30%.

## Discussion

The Title X Program contributes to achieving *Healthy People 2020* objectives for reducing cervical cancer by providing cervical cancer screening to women with low income, many of whom lack health insurance or a regular source of health care. The decline in the percentage of Title X female clients screened for cervical cancer during 2005–2015 is consistent with newer screening guidelines; level or increasing trends in the provision of other recommended preventive services support this observation. The decline in Title X cervical cancer screening, which is based on administrative data, is consistent with downward trends in self-reported screening found in national survey data (*2*,*10*). These data also indicate that self-reported screening rates have declined among females for whom screening was not recommended (<21 years) compared with females for whom the screening interval was lengthened (21–29 years) (*10*).

The findings in this report are subject to at least four limitations. First, FPAR lacks data on cervical cancer screening by age group and type of screening test. This limitation prevents the calculation and analysis of screening rates for younger age groups (<21 and 21–29 years) and for females aged ≥30 years by test type. Second, the aggregate nature of FPAR data prevents a comparison of cervical cancer screening across important client characteristics (e.g., race, ethnicity, income level, or insurance status) or an assessment of whether cervical cancer screening for individual clients is conducted per recommendations or is received elsewhere. Third, the downward trend in cervical cancer screening coincided with a decline in the total number of female Title X clients served by the 64 grantees in this study (4.14 million in 2005 and 3.22 million in 2015) and an increase in the percentage of female Title X clients in the older (≥25 years) age groups. From 2005 to 2015, the percentage of females aged ≤19 years declined from 27% (2005) to 18% (2015) while the percentage of females aged ≥20 years increased from 73% (2005) to 82% (2015). Because of the increased percentage of female Title X clients in age groups for which regular but less frequent (every 3 or 5 years) cervical cancer screening was recommended, the decline in screening might be even more pronounced. According to grantee comments accompanying cervical cancer screening data reported in FPAR (*9*), increased provider adherence to recommendations was a primary reason given for the decline in screening. Finally, during 2005–2015 the number of female Title X clients served by grantees in this study both rose (2005–2009) and fell (2009–2015); in 2015, the number of female Title X clients served was 1.1 million (25%) lower than in 2009. From 2010 to 2015, a 16% decline (by $253.3 million in 2016 constant dollars) in total program revenue (i.e., from Title X and all other sources) reported by all grantees (89 grantees in 2010 and 91 grantees in 2015) was likely an important contributing cause to the decline in number of clients (*9*). Other plausible reasons for the decline in clients include increased use of long-acting contraceptive methods that require fewer visits and health system changes, which might have resulted in some newly insured clients seeking care elsewhere. Aggregate FPAR data are suitable for exploring some but not all of the possible reasons for this decline in clients. 

Aggregate FPAR data allow monitoring of program-level trends in cervical cancer screening. As the Title X Family Planning Program moves forward to replace the current FPAR system with one that will collect client-level and encounter-level data, grantees and subrecipients can use the disaggregated data currently available to examine whether cervical cancer screening performed in their service networks is consistent with recommendations.

**Summary**

**What is already known about this topic?**

Cervical cancer screening is critical to early detection and treatment of precancerous cells and cervical cancer. During 2005–2012, screening guidelines were updated to recommend less frequent screening. In 2015, 83% of women reported being screened according to recommendations. Since 1970, Title X-funded health centers have been a source of cervical cancer screening for primarily socioeconomically disadvantaged women seeking contraceptive and related preventive health care.

**What is added by this report?**

The percentage of female Title X clients screened annually for cervical cancer declined from 51% in 2005 to 21% in 2015 with the largest single-year declines occurring in the years after major recommendation updates (2010 and 2013). Provision of other recommended preventive health services (chlamydia testing and contraception), especially to young females under the recommended starting age (21 years) for cervical cancer screening, increased.

**What are the implications for public health practice?**

The downward trend in Title X cervical cancer screening each year is consistent with current evidence-based recommendations. Aggregate administrative data are useful to describe overall trends in the percentage of Title X clients that received a Pap test. Analyses of client-level and encounter-level records are needed, however, to assess providers’ adherence to screening recommendations and variations in screening practices.

## Footnotes

* “Other” reasons that female clients might not adopt or continue using contraception include 1) the user or her sexual partner either being sterile without having been sterilized surgically or having had a noncontraceptive surgical procedure that has rendered the user or her sexual partner unable to conceive or impregnate, or 2) the user having a sexual partner of the same sex.

† https://www.cdc.gov/reproductivehealth/contraception/mmwr/spr/summary.html.
